# Video directly observed therapy intervention using a mobile health application among opioid use disorder patients receiving office-based buprenorphine treatment: protocol for a pilot randomized controlled trial

**DOI:** 10.1186/s13722-020-00203-9

**Published:** 2020-07-31

**Authors:** Zachery A. Schramm, Brian G. Leroux, Andrea C. Radick, Alicia S. Ventura, Jared W. Klein, Jeffrey H. Samet, Andrew J. Saxon, Theresa W. Kim, Judith I. Tsui

**Affiliations:** 1 Division of General Internal Medicine, Department of Medicine, University of Washington, Mailbox 359780, 325 Ninth Avenue, Seattle, WA 98104 USA; 2 Department of Biostatistics, University of Washington, Seattle, WA USA; 3 Department of Community Health Sciences, Boston University School of Public Health, Boston, MA USA; 4 Clinical Addiction Research and Education (CARE) Unit, Section of General Internal Medicine, Department of Medicine, Boston University School of Medicine and Boston Medical Center, Boston, MA USA; 5 Center of Excellence in Substance Abuse Treatment and Education, VA Puget Sound Health Care System, Seattle, WA USA

**Keywords:** Medication adherence, Mobile health, mHealth, Opioid related disorders, Buprenorphine, Directly observed therapy

## Abstract

**Background:**

Office-based buprenorphine treatment of opioid use disorder (OUD) does not typically include in-person directly observed therapy (DOT), potentially leading to non-adherence. Video DOT technologies may safeguard against this issue and thus enhance likelihood of treatment success. We describe the rationale and protocol for the Trial of Adherence Application for Buprenorphine treatment (TAAB) study, a pilot randomized controlled trial (RCT) to evaluate the effects of video DOT delivered via a smartphone app on office-based buprenorphine treatment outcomes, namely illicit opioid use and retention.

**Methods:**

Participants will be recruited from office-based opioid addiction treatment programs in outpatient clinics at two urban medical centers and randomized to either video DOT (intervention) delivered via a HIPAA-compliant, asynchronous, mobile health (mHealth) technology platform, or treatment-as-usual (control). Eligibility criteria are: 18 years or older, prescribed sublingual buprenorphine for a cumulative total of 28 days or less from the office-based opioid treatment program, and able to read and understand English. Patients will be considered ineligible if they are unable or unwilling to use the intervention, provide consent, or complete weekly study visits. All participants will complete 13 in-person weekly visits and be followed via electronic health record data capture at 12- and 24-weeks post-randomization. Data gathered include the following: demographics; current and previous treatment for OUD; self-reported diversion of prescribed buprenorphine; status of their mental and physical health; and self-reported lifetime and past 30-day illicit substance use. Participants provide urine samples at each weekly visit to test for illicit drugs and buprenorphine. The primary outcome is percentage of weekly urines that are negative for opioids over the 12-weeks. The secondary outcome is engagement in treatment at week 12.

**Discussion:**

Video DOT delivered through mHealth technology platform offers possibility of improving patients’ buprenorphine adherence by providing additional structure and accountability. The TAAB study will provide important preliminary estimates of the impact of this mHealth technology for patients initiating buprenorphine, as well as the feasibility of study procedures, thus paving the way for further research to assess feasibility and generate preliminary data for design of a future Phase III trial.

*Trial Registration* ClinicalTrails.gov, NCT03779997, Registered on December 19, 2018.

## Background

Currently in the United States, there is a crisis of undiagnosed and untreated opioid use disorder (OUD) leading to hundreds of thousands of hospitalizations and drug overdose deaths per year [1]. The CDC estimates that 67.8% of the 70,237 annual drug overdoses in 2017 involved opioids [1]. Drug overdose is one of the major leading causes of adult injury-related deaths in the United States [2]. A 2018 study estimated approximately 2 million Americans live with OUD and about 526,000 live with OUD associated with heroin use [3]. Individuals with OUD who administer opioids via injection are at a higher probability of being exposed to and transmitting infections such as the hepatitis C virus (HCV) and the human immunodeficiency virus (HIV) through sharing and reusing of injection equipment (e.g. syringes, cookers, cottons or rinses) [4, 5]. As such, the rise in use of prescription opioids and phenomenon of transitions to heroin has been tied to recent HIV and HCV outbreaks in urban and rural communities across the United States [6–9]. The current opioid epidemic heightens the need for patient-centered and clinically approved tools and technologies to support treatment for persons living with OUD.

Medications are effective treatment for OUD. Opioid agonist therapy (OAT), namely methadone and buprenorphine, significantly reduces illicit opioid use [10], opioid overdose [11], HIV [12] and HCV infection [13–15]. Buprenorphine, a partial μ-opioid receptor agonist, confers some advantages over methadone, in that it provides less sedation, overdose risk and abuse potential [16]. Many patients prefer buprenorphine over methadone treatment in part, due to greater autonomy related to the system in which it is delivered in the United States (US) [17–19]. The model for office-based buprenorphine treatment does not include in-person daily directly observed medication ingestion, also known as directly observed therapy (DOT), as is standard care for receiving methadone treatment in federally regulated US opioid treatment programs. Instead, patients take buprenorphine at home unmonitored as they would for other medications for chronic diseases. Yet greater autonomy is challenging for some patients who may have imperfect adherence to buprenorphine, which results in worse treatment outcomes (i.e., continued illicit opioid use and lower retention).

Prior research suggests that patients prescribed buprenorphine through an office-based setting do not take, on average, 29% of their medication, and medication non-adherence is associated with illicit drug use [20, 21]. Alternatively, taking more than the prescribed dose can lead to running out of buprenorphine early before the next refill date. For some patients, non-adherence due to missed doses may be the result of buprenorphine diversion [22–24]. One study found that 33% of participants reported giving away, selling, or trading some or all of their prescribed buprenorphine [25]. Greater opportunities for non-adherence and diversion with buprenorphine may contributes to its lower treatment retention than with methadone, which relies on an in-person DOT delivery model [26]; office-based buprenorphine programs retain only approximately 50% or fewer of their patients at 12 months [20, 27, 28]. Over time, patients who do not adhere to buprenorphine in order to use opioids may be lost to follow-up, or providers may discontinue prescribing and/or transfer to a higher level of care due to treatment non-response. Non-adherence and diversion of buprenorphine are provider concerns that drive practice despite the fact that much of diverted medication is used for self-treatment or given to those without direct access [29, 30]. A recent study of providers’ practices to address diversion observed that the majority of providers monitor for non-adherence through urine drug testing (UDT) and a third described diversion as a significant or very significant concern in their community [31].

**Fig. 1:**
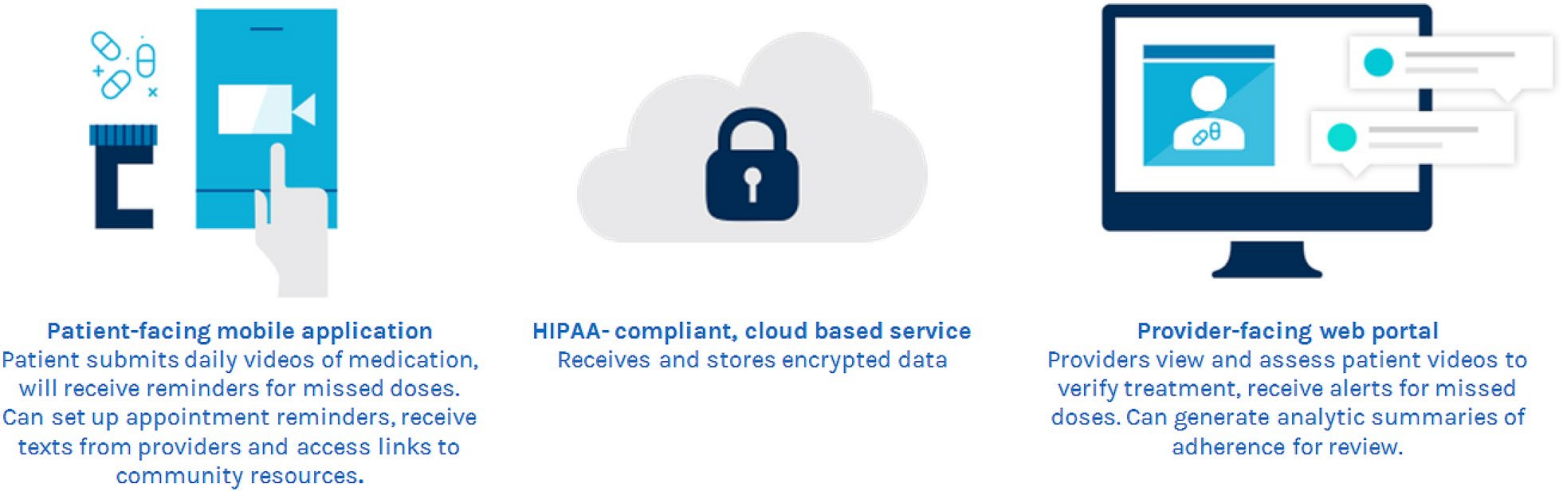
Video-DOT for buprenorphine patient-facing application and provider-facing web portal functionalities

Mobile health (mHealth) technologies hold potential to promote accountability and structure for patients on buprenorphine, and address providers’ concerns by potentially acting as a safeguard against non-adherence and diversion [32–36]. According to the World Health Organization, mHealth consists of “medical and public health practices supported by mobile devices, such as mobile phones, patient monitoring devices… (and) involves the use and capitalization on a mobile phone’s core utility of voice and short messaging services as well as more complex functionalities and applications…“ [37]. Video DOT utilizes the front facing camera of mobile devices to confirm proper medication ingestion and adherence. Video-based mobile technology for medication adherence has been successfully implemented in the treatment of active tuberculosis, cutting back on the need for face-to-face visits for monitoring [38–40]. Similar to treatment for tuberculosis, video DOT would obviate the need for directly observed, in-person administration of buprenorphine. A video DOT mobile application could be “prescribed” by a provider to patients who may struggle with adhering to buprenorphine. Such a mHealth application may promote medication adherence by sending medication reminders and notifications to patients when no videos have been submitted. The reviewed videos provide a system of accountability that can be utilized by patients and providers (Fig. 1).

**Fig. 2:**
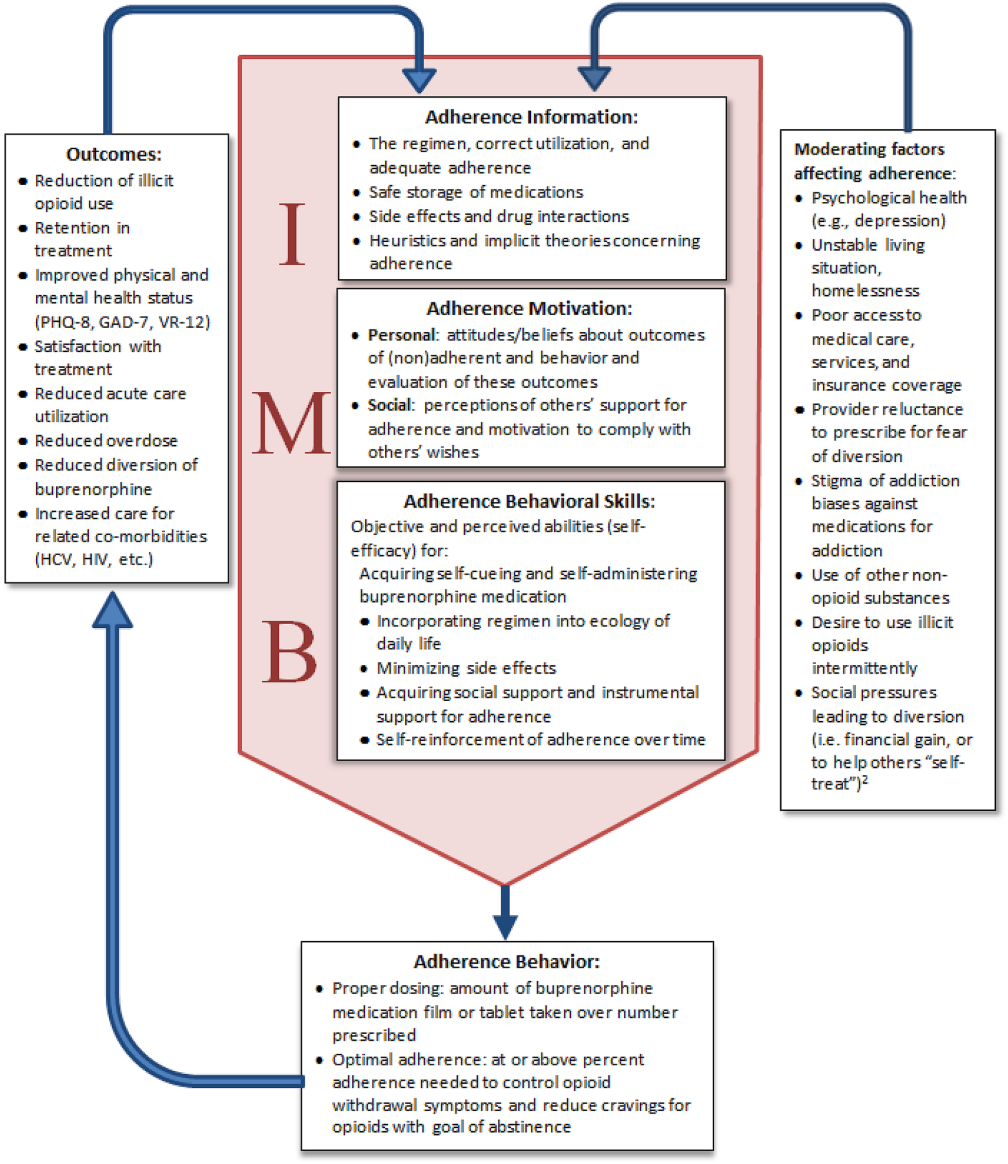
Information-Motivation-Behavior Skills (IMB) model for buprenorphine adherence targeted by video-DOT in an office-based setting 1. Adapted from the Information-Motivation-Behavioral Skills (IMB) Model of HIV medication adherence (Fisher J. D., Fisher W. A., Amico K. R., Harman J. J. An Information-Motivation-Behavioral Skills Model of Adherence to Antiretroviral Therapy. Health Psychol. 2006;25 [4]:462–73. 10.1037/0278-6133.25.4.462. PubMed PMID: 16846321.). 2. Schuman-Olivier Z, Albanese M, Nelson SE, et al. Self-treatment: illicit buprenorphine use by opioid-dependent treatment seekers. J Subst Abuse Treat. 2010;39 [1]:41–50

Qualitative research examining perspectives of patients receiving buprenorphine and their prescribing providers on the acceptability of video DOT for buprenorphine treatment found that most patients had a favorable impression, affirming that such a tool could help promote accountability and patient/provider trust [32]. Participating providers also thought that it could be particularly helpful for patients who are newly engaged in treatment and specifically to prevent diversion. However, social and structural barriers to treatment adherence, such as homelessness, chaotic lifestyles, mental illness, and limited access to or understanding of technology, were perceived as ongoing challenges to its implementation that would need to be addressed. This initial qualitative study helped researchers identify useful in-app features and the feasibility of daily video DOT, which facilitated the development of an early application prototype used for a single-arm feasibility study of 14 patients receiving buprenorphine treatment. The feasibility study found that nearly all (13/14; 93%) were able to use the application successfully to upload videos [33]. Based on this prior research, we developed a conceptual model for how video DOT might affect buprenorphine adherence in an office-based setting (Fig. 2). This model was adapted from the Information-Motivation-Behavioral Skills (IMB) Model of HIV medication adherence [41–44]. We conceptualize that challenges to buprenorphine adherence can fall within the three IMB domains of information, motivation and behavioral skills, and we postulate that offering a video DOT mHealth intervention can address certain challenges in each of these domains of adherence which ultimately results in better treatment outcomes (i.e., reduced illicit opioid use and better retention).

Here we describe the protocol for the Trial of Adherence Application for Buprenorphine treatment (TAAB) study, a pilot randomized controlled trial (RCT) to evaluate the effects of a video DOT mHealth application on buprenorphine treatment outcomes. The purpose of this pilot trial is to assess feasibility and generate preliminary data for design of a future Phase III trial.

## Methods

### Study design

The TAAB study is a two-site pilot randomized controlled trial of a behavioral mHealth intervention. Participants will be randomized to one of two arms; treatment-as-usual (TAU) (control) or TAU + video DOT (intervention) delivered via mobile smartphone, with a 1:1 allocation ratio.

### Setting and participants

Study participants will be recruited from office-based opioid treatment (OBOT) programs in primary care and psychiatry clinics at two urban medical centers in Seattle, WA and Boston, MA. The Seattle site consists of OBOT programs in adult primary care and psychiatry, while the Boston site includes a program in adult primary care. Programs at each site offer similar models of office-based buprenorphine treatment based on the Massachusetts Collaborative Care Model which utilizes nurse care managers (NCMs) to assist buprenorphine-waivered physicians and advance practice practitioners, details of which have been previously published [45, 46]. The NCMs serve as the “hub” for patient care; they take the bulk of responsibility for conducting visits, ensuring scripts are written, referring patients to needed services (such as for counseling, housing, transportation and employment) and conducting urine drug tests. For this pilot study, participants randomized to TAU only received care delivered via this care model which included weekly and/or biweekly visits with NCMs or providers. Those assigned to the intervention continued to receive this standard level of medical care in addition to receiving the video DOT mobile health application.

Eligibility criteria are the following: 18 years or older, within their first 28 days of either starting or restarting prescribed sublingual buprenorphine treatment from office-based treatment program recruitment sites, and able to read and understand English. Treatment “restarts” are deemed so by the provider, and generally occur in the setting where a script has lapsed for more than a month. Patients will be deemed ineligible if they are unable or unwilling to use the mobile application, are cognitively impaired and unable to provide informed consent, have immediate plans to move such that they cannot complete study visits, or are aware of imminent incarceration.

Potential participants will be identified by querying the electronic health record (EHR) and clinic schedule, recruitment flyers posted at recruitment sites and referrals from clinic staff. Study staff approach patients after their first clinic visit or during subsequent follow up visits at the clinic within the patients’ first 28 days of treatment to introduce themselves and the study. Nurses inform patients that research staff are present to assess patient interest in participating in a study of a mobile phone application aimed to improve buprenorphine adherence. If patients verbally confirm interest to meet with staff, nurses provide a “warm hand-off” so that the research staff and patient can discuss the study in private. Staff will administer the study eligibility screener via the Research Electronic Data Capture (REDCap) web platform to those who verbally consent to be screened and, if eligible and willing to participate, complete consent, enrollment and randomization along with the baseline questionnaire. Patients can defer meeting with staff for enrollment to a later date after screening yet must enroll in the study before their first 28 days of buprenorphine treatment. Consenting, randomization and baseline occurs on the same day and group assignment will be disclosed after the completion of the baseline visit. Eligible and interested patients will provide informed consent and then be randomized to the mHealth application or treatment-as-usual. Randomization is stratified by site and blocked with random block sizes of 2, 4, 6, or 8 to ensure concealment. Participants will be informed of their group assignment after the completion of their baseline visit. The TAAB study received approval from the University of Washington’s Institutional Review Board (IRB) in Seattle, Washington and from Boston Medical Center and Boston University Medical Campus Institutional Review Board in Boston, Massachusetts.

### Study procedures and data collection

After the baseline research visit, participants will complete 12 in-person weekly visits. The components of data collected at each study visit are summarized in Table 1. At baseline, participants will report on their demographics; current and previous use of medication for OUD (methadone or buprenorphine); history of buprenorphine diversion (“*Have you ever taken buprenorphine/naloxone in any way other than placing it under your tongue?”* and *“Have you ever sold, gave away, traded, lent or lost any buprenorphine/naloxone that was prescribed to you?”)*; status of current mental and physical health via the Veteran-RAND 12 (VR-12) [47], depression via the Patient Health Questionnaire 8 (PHQ-8) [48], anxiety via the Generalized Anxiety Disorder 7 (GAD-7) [49]; and lifetime and past-30 day illicit substance use via the Addiction Severity Index (ASI) [50]. At each in-person study visit, adherence to buprenorphine for the past seven days is to be assessed utilizing a modified calendar timeline follow-back (TLFB) procedure similar to prior studies [51, 52]. Adherence to buprenorphine per self-report is defined as taking the correct amount of medication as prescribed (i.e., the exact dose) for each day over the past seven days. Participants who may report taking a total daily dose that is higher, lower or no daily amount of buprenorphine than prescribed in any of the past seven days will be considered to be non-adherent. Participants reporting splitting total daily doses into two or three doses throughout their day are considered adherent as long as their self-report equals the prescribed daily total of buprenorphine. Additionally, participants who report taking prescribed buprenorphine in any other way than sublingually will be considered to be non-adherent. EHR will indicate the dosage that the patient’s script was written for, yet it cannot be assumed that patients take their medication as prescribed. Splitting doses may be a personal preference which may not be reflected in the script, and also some patients will take less or more medication than directed. Furthermore, providers may verbally give instructions to modify a dose after a script has been written without making changes to the EHR.

**Table 1:**
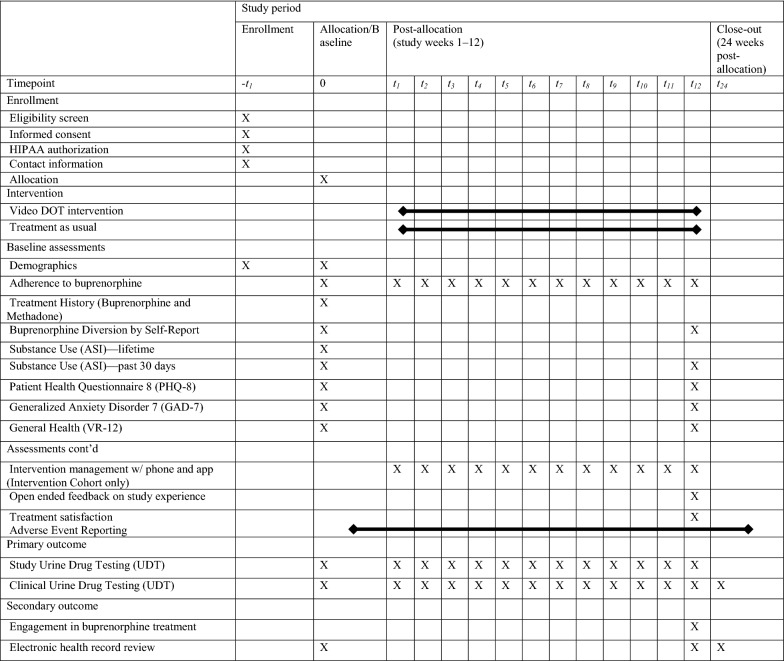
Summary flowchart of the TAAB study schedule of enrollments, interventions, and assessments

Participants will also provide a urine sample at each weekly follow-up research visit to test for the presence of other illicit drugs including non-prescribed medications as well as to verify recent buprenorphine use. Only research staff members will be aware of the results of research UDT; study participants will not be informed of test results and results will not be available to clinical staff. The frequency of clinically ordered UDT is determined by providers according to program policies which differ between sites. At the final visit, all participants will be asked again about changes in buprenorphine dose, any buprenorphine diversion during the 12-weeks, mental and physical health via VR-12, PHQ-8, and GAD-7, illicit substance use over the past 30 days via ASI, and their satisfaction with the treatment they received from their clinic. Participants will finally be asked open-ended question(s) related to their experience in the study and the intervention (Appendix 1). All participants will be compensated $50USD for completing baseline and final visits, and $20USD for completing each weekly follow-up visit. Intervention participants will be compensated for attendance at study visits regardless of whether videos are uploaded.

**Fig. 3:**
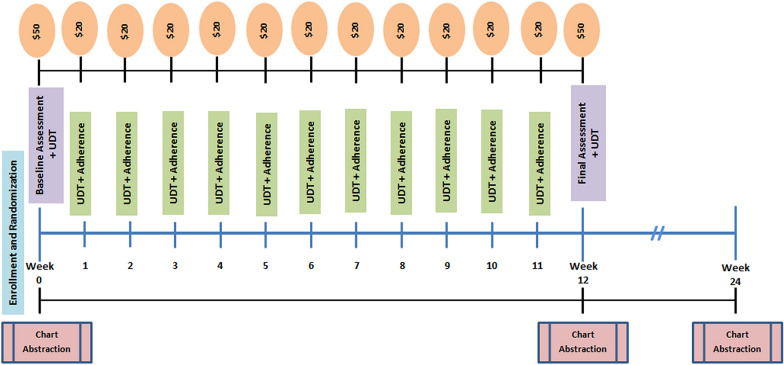
Outline of study procedures provided to participants during the informed consent process

In addition to participant interviews, research staff will review the EHR to assess continued receipt of treatment in the OBOT program, noting any changes such as discontinuation from the program, treatment interruptions due to incarceration or hospitalization, or if there is change in treatment (such as transfer to a methadone program or in-patient residential treatment). Results of UDTs performed by the clinic will also be collected if information from a research study UDT is not available. These reviews will look at data from the time of enrollment at baseline, 12- and 24- weeks post enrollment. Figure 3 was developed by research staff to help participants understand the study procedures and incentives timeline and will be included in the informed consent process.

Staff will report any event that meets the definition of an unanticipated problem to their respective IRB and appropriate oversight bodies within the timeframe that is required. All AEs and SAEs that do not meet the definition of an unanticipated problem will be collected and reported in aggregate to the Data Safety Monitor for independent review. Staff will report on any event that meets these definitions from the time of enrollment thru week 24 post-enrollment.

### Study intervention

The study intervention is a mHealth intervention which enables video DOT of buprenorphine treatment via mobile devices, primarily via smartphones specifically but could be used via tablet devices as well. The application was developed by emocha Mobile Health®. The emocha mobile video app is part of a HIPAA-compliant, asynchronous, video-based technology platform which facilitates video DOT. Many of the study intervention features address key points of the modified IMB model for buprenorphine treatment adherence (Fig. 2). For example, intervention participants will have informational links to institution-specific patient portals, local community and social support groups, and clinic treatment phone numbers and resources as well as specific buprenorphine medication treatment information located within the mobile application intervention. These features highlight the importance of having access to medication and treatment information as well as social and community resources that help develop motivation to adhere to treatment in addition to the video DOT feature of the application.

Intervention study participants who have a personal smartphone will install the video DOT application on their smartphone or tablet. Participants who do not have or are unwilling to use their personal mobile device will be offered a study smartphone with the application installed (included cellular shared data plan). The application works on both Android and iOS systems. The study will provide up to one replacement smartphone if damaged, lost or stolen. Participants will not be incentivized to return the phone at the end of the 12 weeks. Those who fail to return two study phones will not receive a third but will be able to continue to be involved in the study and meet with staff for remaining follow-up visits.

**Fig. 4:**
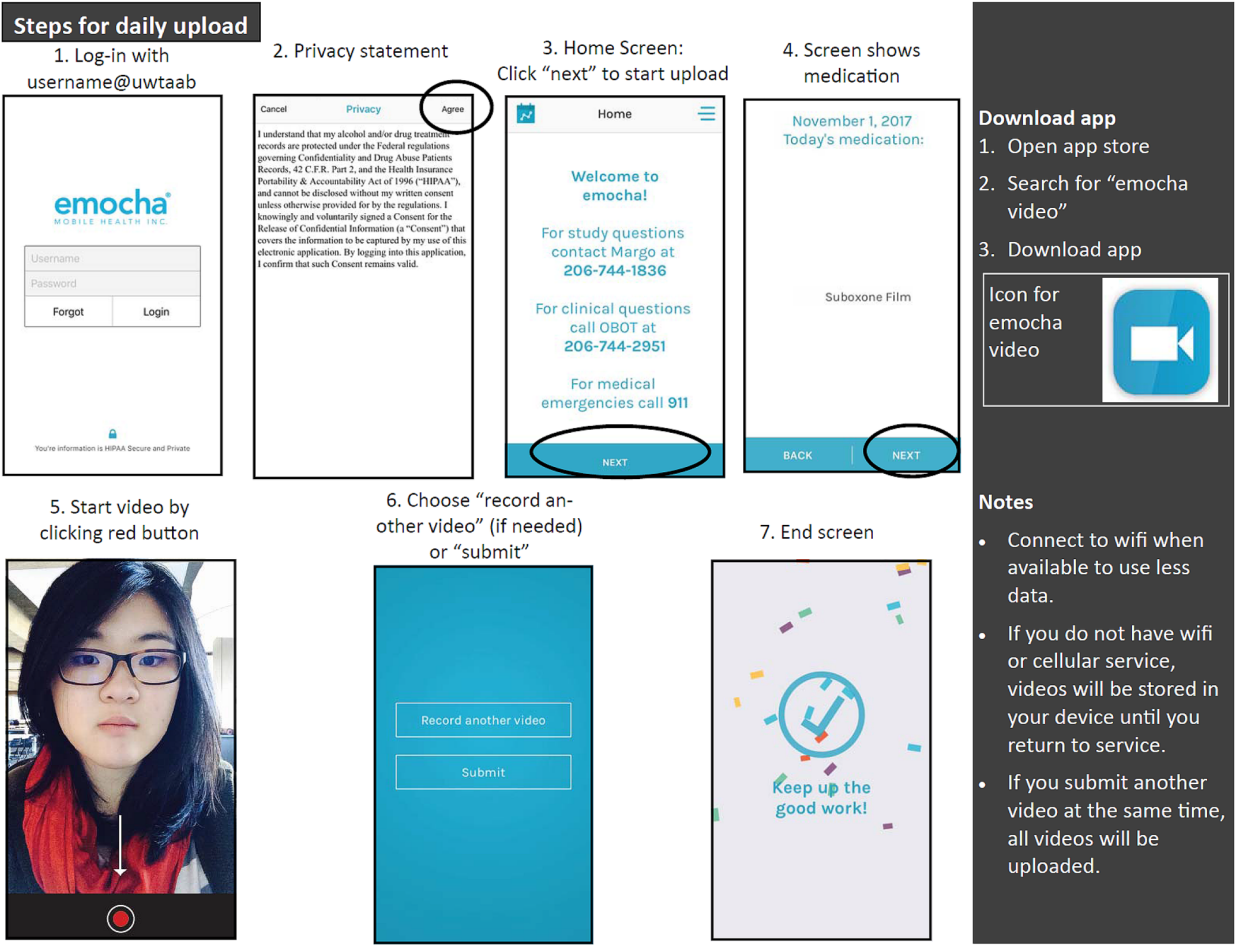

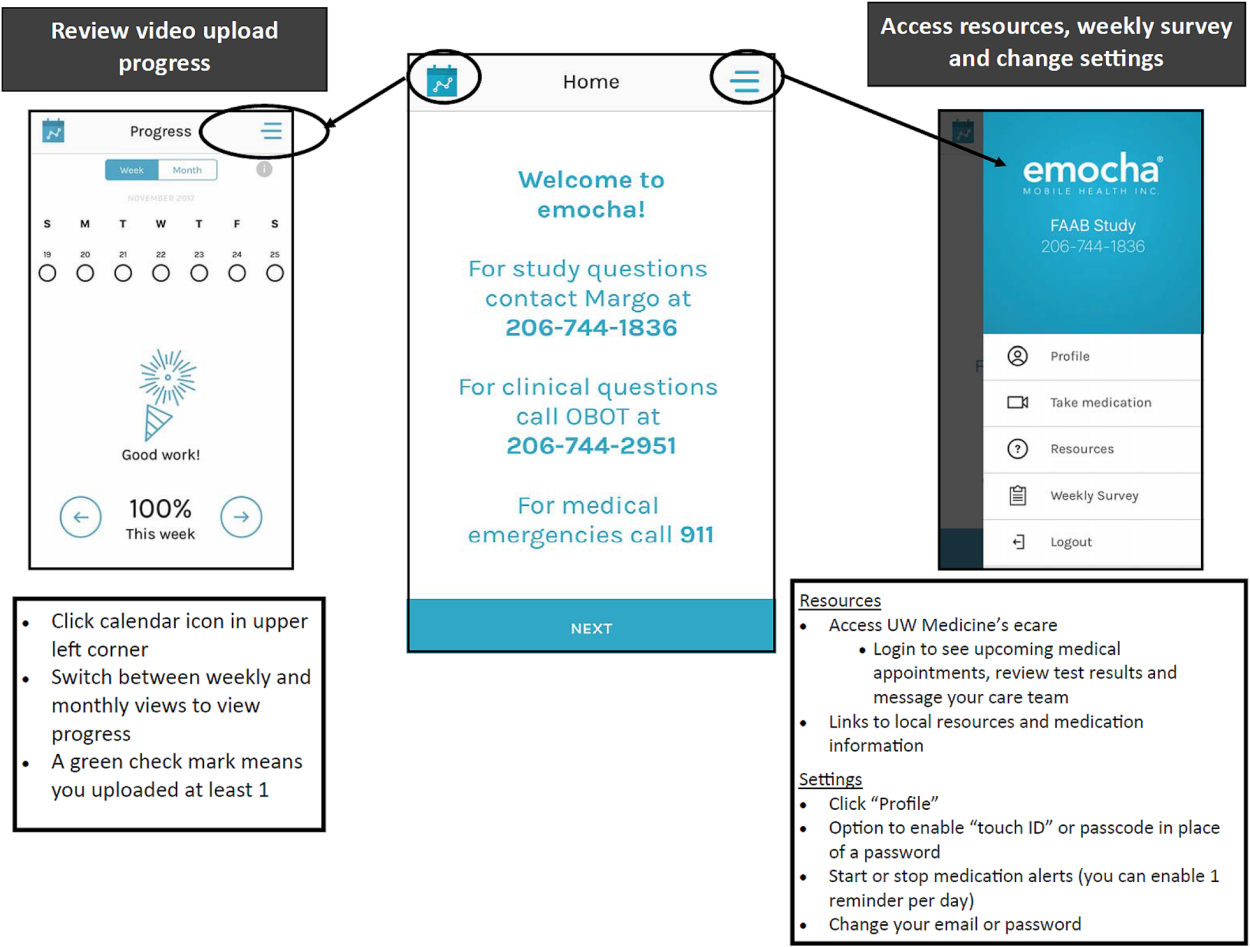
Instructions explained and provided to intervention participants on steps needed to upload daily videos

Following the completion of the baseline visit, a research staff member will create an account for the newly enrolled study participant on the HIPAA secure web-based provider portal, provide information regarding in-app features including how to properly upload videos (Fig. 4), and review security information related to their videos. Participants will use the mobile application to video record themselves taking their buprenorphine medication. Participants will be asked to (1) present the medication on the screen, (2) show the placement of the medication sublingually, and (3) continue to record for 3 min. Although sublingual buprenorphine will take longer to be completely dissolved, 3 min will be encouraged to minimize burden on participants; however, submitted videos that are less than 3 min but longer than 1 min will be considered acceptable. Participants will be informed to remain in the frame of the video for the requested 3 min. Research staff will suggest that participants give an update on their day, read a book or newspaper, or simply take the time to relax for a moment. At the time of consent, participants will agree to avoid including other individuals or behaviors that they do not wish researchers or providers to see in the frame, as certain behaviors may require reporting to authorities (such as attempts to harm others or self). While participants will be encouraged to video all buprenorphine doses, the study definition for adherence to using video DOT is defined as uploading at least one video a day for 12 weeks regardless of whether or not the participant was prescribed two or more times per day. As such, the objective of the intervention is not to necessarily safeguard against diversion completely, rather to create a “user-friendly” intervention that would promote at least daily adherence to medication. The decision to require only a single video was based on information gained from a prior pilot study which demonstrated that submitting multiple videos a day had low acceptability to participants [33].

Finally, intervention participants receive a tutorial of all details on features and settings of the application and their account. Participants will have the ability to edit medication dosing reminders, including selecting not to receive reminders. Default daily reminders saying “Please remember to take your medication” are sent at 8:00 AM. If no video is uploaded by 4:00 PM another reminder is sent. Participants will have the ability to alter the timing of medication video DOT reminders as well as the form, short messaging services (SMS) or in-app push notifications, and frequency of the reminders. This reminder feature serves to address the development of self-cueing and self-administration of medication thus further developing key behavioral skills necessary to incorporate medication into their daily routine, a key tenement of the IMB model. Participants can retrieve a calendar summary of their weekly and monthly adherence in the emocha app and see the status of their submissions (waiting review, accepted, rejected, missed, etc.) creating a motivational information feedback loop regarding proper utilization of the application for adherence as well as providing medication adherence tracking on whether medication was taken that day or not.

Several features of the emocha mobile video app serve to protect participants’ privacy regarding their medical treatment. All videos will be digitally encrypted and stored temporarily in a secure file location in the device’s hard drive. Video play-back on participants’ device is not possible. Access to the mobile app is password-protected, so participant medication and adherence calendar data will not be accessible to other individuals who may use the participant’s mobile device. Encrypted videos will be sent to the app developer’s HIPAA secure servers when the phone is connected to either a cellular or other wireless network. In-flight encryption ensures that data remains encrypted and protected while being transmitted. Data being sent from the app is sent over a secure HTTPS connection secured by a 2048-bit SSL certificate. This allows for security even when using the application on unsecure (i.e., non-password protected) wireless internet. The emocha app may be used offline and transmit encrypted videos once a network connection is restored. Once successfully uploaded, videos are erased from the participant’s device. A research staff member will review the videos asynchronously on the provider web portal to decide whether the video meets all three specifications for an acceptable medication adherent video. Staff will review video submissions frequently, if not daily; yet with holidays and weekends, participants will be notified that it may take up to 3 days before a video is reviewed. This is to allow for the most up-to-date representation of analytics on the participant’s in-app calendar tracking feature. The emocha app does not allow for synchronous (i.e., real-time) review of medication adherence. Videos will be rejected if (1) the video quality is poor (i.e., low lighting) resulting in the inability to recognize the study participant or observe proper medication adherence, (2) there is no medication viewable in the video, or (3) the video is less than one minute. The 1 min minimum for acceptable videos starts after the placement of buprenorphine medication sublingually. Participants will be instructed to refrain from recording other things (such as behaviors or other individuals) that they do not wish research staff, mHealth application developers, and clinicians to view. When staff review videos and find that they meet all specifications, they are to click “accept” thus documenting the video as acceptable on the portal and within the underlying data set of the provider portal.

In the first 2 weeks of intervention usage, research staff members will identify early issues with proper video adherence and provide active feedback to the participant on their past week patterns of video uploads. This may include reminding participants to record for 3 min daily or to show medication prior to placement in the mouth. There is no intervention protocol to provide support to participants who are not adhering to submitting videos as this study is conducted to further understand if and how often patients may be willing to submit medication adherence videos without any built-in contingencies, incentives, or additional intervention. However, research study staff will continue to query intervention participants at each weekly follow-up visit about any difficulties encountered using the phone or mHealth application. Specifically, research staff members will ask intervention participants if they experienced any difficulties using the application in the past week. If participants disclose any issues, the research staff member documents it and then will attempt to address it with the participant to resolve the issue. Research staff will also let participants know that they are available for any questions by phone, or in-person at weekly visits, throughout the study.

The prescribing clinicians and NCMs will be provided access to the provider web portal and given the option to review their participants’ videos and summaries through the portal, although this will not be required. However, providers all will receive a hard-copy monthly summary of the participant’s video DOT adherence (Appendix 2). Participants will be encouraged to review their video DOT adherence calendars with providers at in-person clinic visits, like how a patient with diabetes might review their glucometer results with a provider.

### Data management and monitoring

All data from eligibility screenings, research visits, EHR reviews, and adverse events will be collected via REDCap. Access to the REDCap servers is provided by the University of Washington’s Institute for Translational Health Sciences. Data will be protected by using unique study IDs and stored in password protected computers and programs with only trained research staff having access. Identifiers needed to track participants will be kept separate from research data. All videos uploaded by video DOT participants at both sites are encrypted and stored in separate site-specific HIPAA compliant web-based emocha entities. Only approved research staff members and providers will have access to their corresponding site web portals. Research staff members meet biannually with an Independent Safety Monitor to review the safety of participants, collected adverse events and the validity and integrity of the data. Adverse events will be reported in the main results paper.

### Outcomes and measures

The pre-specified primary outcome of interest is percentage of weekly UDTs that are negative for opioids. The main hypothesis is that participants in the video-DOT arm will have a higher percentage of weekly UDTs negative for opioids compared to TAU during the 12-week intervention period. The rationale for choosing opioid negative urine results was to determine if video DOT achieves the desired health outcome from receiving office-based buprenorphine treatment, namely reduction of illicit opioid use over time. The secondary pre-specified outcome of interest is engagement in treatment at week 12, the hypothesis being that participants in the video-DOT arm will be more likely to be engaged in office-based treatment with buprenorphine at the conclusion of the 12-week intervention period compared to participants who were randomized to TAU. This is aligned with the modified IMB model where adherence is an intermediate outcome which leads to better treatment outcomes (less illicit opioid use and improved retention). Although adherence was considered as a primary outcome, it was felt that a future study of efficacy would need to demonstrate improvement in distal outcomes, particularly since DOT is not currently the standard of care for buprenorphine.

To test the primary study hypothesis, point-of-care urine drug tests will be administered at weekly research visits for all participants. Testing will be conducted with the Alere 14 Drug Panel iCups® which test for 14 substances and their metabolites (cannabis, cocaine, morphine, oxycodone, methadone, benzodiazepines, barbiturates, amphetamines, methamphetamines, nortriptyline, 3,4-methylenedioxymethamphetamine (MDMA/ecstasy), phencyclidine, and propoxyphene). Additionally, we will assess for the presence of fentanyl in the urine using fentanyl test strips which are at a preset 300 ng/ml cutoff level, consistent with the Substance Abuse and Mental Health Services Administration’s (SAMSHA) immunoassay test guidelines. If collected by clinicians, weekly clinical UDT results will be recorded from the EHR following the completion of weekly research visit. Additional prescription medication information related to prescribed opioids, benzodiazepines, and stimulants will be collected at the 12-week post-enrollment EHR review. This data will help to corroborate any positive study UDT results that should not be considered illicit substance use.

To test the secondary study hypothesis, information from the EHR will be obtained regarding the participant’s buprenorphine treatment at 12 weeks post-randomization, specifically whether participants are still receiving prescribed buprenorphine medication. Treatment engagement is defined as having an issued covering script for buprenorphine that has been active within the prior 7 days at the time of the week 12 post randomization EHR review date. Additional analyses will be conducted to confirm and validate participants’ self-report of continued buprenorphine treatment at the final in-person study visit.

Exploratory outcomes are: (1) engagement in treatment at week 24 post-enrollment; (2) time to end of engagement in treatment (censored at 24 weeks for those engaged at week 24); (3) number of consecutive weeks with study urine drug test negative for opioids during the 12-week study period; (4) self-report of days of use of illicit opioids in the past 30 days assessed at week 12; (5) percentage of days adherent to buprenorphine by weekly TLFB self-report during the 12-week study period; (6) having one or more study urine drug test negative for buprenorphine any time during the 12-week study period; (7) having a study urine drug test positive for stimulants (cocaine, amphetamines or methamphetamines) at week 12; and (8) patient satisfaction with treatment at week 12.

### Statistical analysis plan

The purpose of this pilot trial is to assess feasibility and generate preliminary data for design of a future Phase III trial. The target sample size of 80 was selected in order to provide sufficient information from both sites (40 per site) on study feasibility and estimates of parameters needed for design of future studies. Based on results from the buprenorphine maintenance therapy arm of a published 14-week trial [53], we anticipate that the percentage of opioid negative UDT will be approximately 50% in the TAU arm. This is similar to the results of our pilot feasibility study, in which 42.9% had a urine drug test positive for opioids at baseline [33]. This current study is not fully powered to test the null hypothesis of no difference between arms in proportion of opioid negative urine samples. Power was estimated to be 69% to detect a difference of 20% between the percentages of negative samples in the two treatment arms.

To evaluate the primary hypothesis, we will fit a log-linear regression model to the weekly UDT test outcomes using Generalized Estimating Equations (GEE) to account for correlation between outcomes within participants, with adjustment for study site. The GEE method can incorporate multiple observations from each subject and is in general more efficient than analyses using a single time point, which means that it can detect smaller differences between groups. The method yields approximately unbiased estimates of regression coefficients, allows the incorporation of subjects with partial information, and accounts for clustering within subject using robust (“sandwich”) estimates of standard errors without a need to correctly specify the intracluster correlation structure. The results of this analysis will be expressed as a percentage increase (or decrease) in the proportion of negative UDT for video DOT compared with TAU, with a 95% confidence interval. Analyses will be done according to the intent-to-treat principle which includes all randomized participants, to be analyzed according to random assignment, regardless of their treatment adherence. Additional “per-protocol” analyses will be conducted based on actual rates of intervention usage rather than assignment to intervention arm as sensitivity analyses.

To evaluate the secondary hypothesis, we will use Poisson regression with robust standard errors to compare treatment groups on the proportion of participants engaged in treatment at 12 weeks (i.e., still receiving medication). A Poisson regression model will be used in order to provide an estimate of treatment effect expressed as a risk ratio for video DOT compared with TAU. The analysis will be done according to the intent-to-treat principle and additional “per-protocol” analyses will also be conducted.

For all analyses we will calculate 95% confidence intervals for treatment effects which will be used to guide sample size calculations for future studies. The intra-class correlation coefficient (correlation between outcomes for urine samples from the same participant) will be estimated for use in power calculations for future studies. We will also fit random effects Poisson regression models and compare the results with those obtained using GEE. This will allow us to determine if there are efficiency gains possible from using random effects models and whether their assumptions are valid for this type of data. Additional analyses will be conducted to estimate parameters of the distributions of outcome variables in the control group, with confidence intervals. These are critical along with estimates of treatment effects for design of a future Phase III trial. We will also explore associations between outcomes and participant demographics and other characteristics for consideration as possible inclusion/exclusion criteria or stratification variables for future trials. Missing data patterns will be explored to assess the potential impact of missing data on bias of parameter estimates and power for tests of treatment effects and inform design and analysis plans for future studies.

## Discussion

This paper describes the design and protocol for the TAAB study which is a pilot RCT of patients - prescribed sublingual buprenorphine for OUD in an office-based setting comparing a video DOT delivered via a mHealth platform to TAU. The study will be conducted to assess the feasibility of research procedures and to generate data to guide design of future studies using video DOT delivered via a mHealth platform.

This study will provide valuable contributions to the rapidly growing field of smartphone-delivered addiction treatment tools. Mobile health technologies are being widely developed and implemented to improve treatment for a variety of other chronic medical conditions [35, 36]. A number of innovative mobile phone applications have been tested for substance use disorders [54–57] and OUD, specifically [54–56, 58, 59]. In our previous single-arm pilot study [33] mHealth delivered video DOT for buprenorphine appeared to be feasible and acceptable. The current study represents an important next step to establish whether use of the technology shortly after buprenorphine initiation can lead to better OUD treatment outcomes, namely less illicit opioid use and improved treatment retention. We hypothesize that the mHealth video DOT intervention may lead to better adherence, and thus improved treatment outcomes, through a number of pathways that impact motivation and adherence behavioral skills (Fig. 2). Yet this intervention, as with other mHealth interventions, needs more evidence before it can be recommended and widely implemented as an adjunct to office-based buprenorphine treatment [60, 61].

The innovative nature of the study intervention can be perceived as a strength. We are unaware of any prior published studies of video DOT via smartphone for buprenorphine alone without incentives to upload videos, although its use has been applied toward treatment of other diseases [40, 62–64] and video DOT for buprenorphine with financial incentives is currently being investigated [65]. The randomized, controlled study design is a study strength, as is the enrollment at two different sites which enhances generalizability of results. Study participants early in treatment, while often challenging to study due to many existing social challenges such as homelessness, active substance use, and mental health co-morbidities, are a vulnerable population for which there is a compelling need to find and rigorously investigate interventions to improve health outcomes. The study has chosen clinically relevant outcomes (i.e., illicit opioid use and retention) that are measured in a rigorous fashion (i.e., UDT) to investigate the study intervention effects.

There are important limitations of this study to consider. First, with a small sample size and limited power to detect meaningful differences we cannot make definitive conclusions on estimates of effect size for our outcomes but can still provide data and a knowledge base needed to effectively develop a larger trial. The study is conducted in two primary care office-based buprenorphine programs that both utilize a nurse-care manager model for collaborative care. While utilizing two sites should provide more evidence of generalizability compared to a single site, results may not generalize to all settings. TAU for this study may be better than the standard of care in other buprenorphine programs that do not use this model, and this could limit our ability to demonstrate and generalize a benefit. Additionally, collected clinical UDT evaluated the same drugs as the study UDT, yet given that there were two sites using different clinical UDT assays the thresholds of detection may differ for some tests. The study will not provide incentives for participants to use the intervention, therefore actual rates of uptake of the intervention may be low which could bias our results to the null. We considered a study design which would link submission of videos to financial incentives. However, we ultimately decided against this as our primary goal was to test the effect the video DOT alone. Since contingency management has been found to be beneficial in treatment for OUD [66], it would be difficult to disentangle the effect of video DOT from the effect of financial incentives. Also, video DOT with financial incentives might be less feasible to implement in most real-world settings. An additional limitation is limited involvement of providers and clinic staff in the intervention. We initially considered a study design which would utilize providers to review videos for acceptability. However, qualitative work revealed that providers felt that this activity was outside their scope of practice and too time-consuming without a system in place for financial reimbursement (which did not exist at the time). Therefore, we chose to utilize research staff to review videos and providers were instead provided summaries of their participants’ video adherence. Finally, our adherence definition is based on submission of at least a daily video: this was deemed appropriate based on experience from a prior feasibility study which demonstrated that requiring submission of multiple videos per day was too burdensome to participants. While this decision was necessary from a practical standpoint, it means that we will be unable to confirm all doses for participants who split their daily dose. Still, confirmation of any doses represents an improvement over standard of care, which is not to require any confirmation beyond urine drug tests which only reflects immediate use prior to testing.

## Conclusion

The Trial of Adherence Application for Buprenorphine treatment (TAAB) study is a pilot randomized controlled trial to evaluate the effects of a video DOT mHealth application on buprenorphine treatment outcomes. This paper describes a framework for conceptualizing how video DOT may impact buprenorphine treatment adherence and the procedures to measure how this technological intervention may ultimately affect clinically meaningful outcomes such as illicit opioid use and retention. Given the growing interest in mobile applications in the treatment of substance use, many of which have not been tested, the study design and protocol presented can guide future research to determine the efficacy of video DOT and other mHealth interventions as an adjunct for buprenorphine treatment.

### Trial status

This trial completed enrollments following the initial submission of this manuscript and data collection is ongoing at the time of resubmission.

## Figures and Tables

**Fig. 5:**
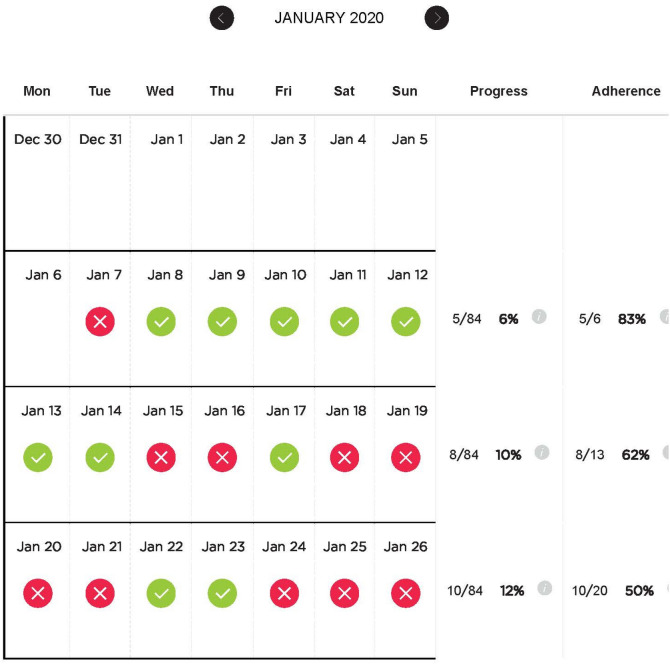
Participant emocha adherence calendar (Page 1)

**Fig. 6:**
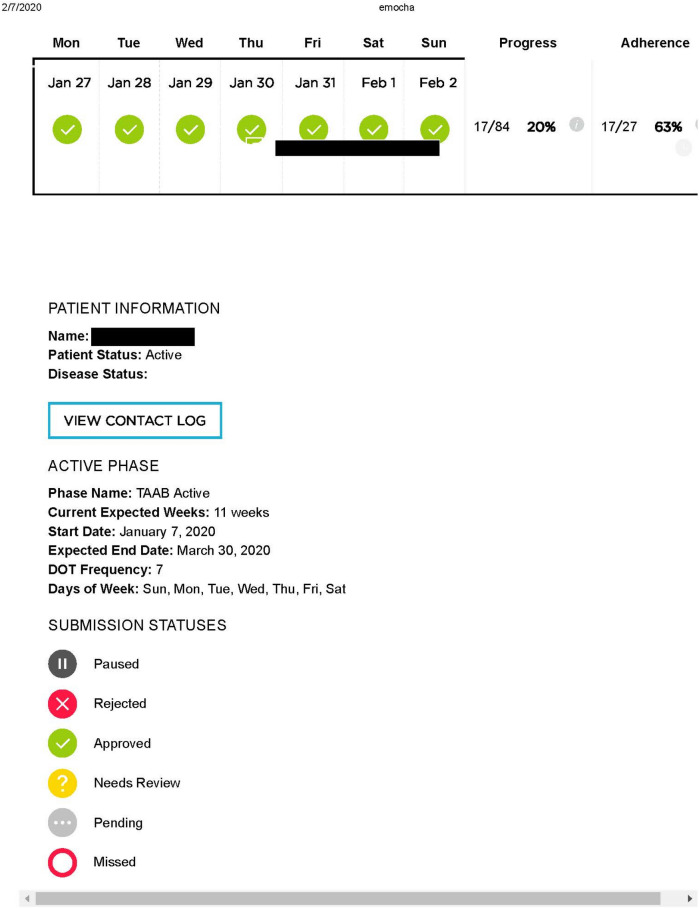
Participant emocha adherence calendar (Page 2)

## Data Availability

The datasets used and/or analyzed during the current study are available from the corresponding author on reasonable request.

## Appendix 1

TAAB Study Final Visit Open-ended Questions

### TAU + video DOT and TAU arms

“How was your experience participating in this study?”

### TAU + video DOT only

“What did you like best about the emocha app?”

“What did you like least about the emocha app?”

“What, if anything, made the emocha app hard or easy to use?”

“Did emocha have any impact on your relationship and interactions with your providers (nurses or doctors)? If so, how?”

“In regards to the emocha application, do you have any recommendations for changes?”

## Appendix 2

See Figs. 5 and 6.

## Abbreviations

OUD: Opioid use disorder
HCV: Hepatitis C Virus
HIV: Human immunodeficiency virus
OAT: Opioid agonist therapy
DOT: Directly observed therapy
UDT: Urine drug test
mHealth: Mobile health
IMB: Information, motivation and behavioral skills
TAAB: Trial of adherence application for buprenorphine
TAU: Treatment-as-usual
RCT: Randomized controlled trial
OBOT: Office-based opioid treatment
NCM: Nurse care manager
EHR: Electronic health record
REDCap: Research electronic data capture
IRB: Institutional review board
VR-12: Veteran-RAND 12
PHQ-8: Patient Health Questionnaire 8
GAD-7: Generalized Anxiety Disorder 7
ASI: Addiction severity index
TLFB: Timeline follow-back
SMS: Short messaging services
SAMSHA: Substance Abuse and Mental Health Services Administration
GEE: Generalized estimating equations
